# Open-Vocabulary Predictive World Models from Sensor Observations

**DOI:** 10.3390/s24144735

**Published:** 2024-07-21

**Authors:** Robin Karlsson, Ruslan Asfandiyarov, Alexander Carballo, Keisuke Fujii, Kento Ohtani, Kazuya Takeda

**Affiliations:** 1 Graduate School of Informatics, Nagoya University, Nagoya 464-8603, Japan; fujii@i.nagoya-u.ac.jp (K.F.); ohtani.kento@g.sp.m.is.nagoya-u.ac.jp (K.O.); kazuya.takeda@nagoya-u.jp (K.T.); 2 Independent Researcher, 1005 Lausanne, Switzerland; ruslan.asfandi@protonmail.com; 3 Department of Electrical, Electronic and Computer Engineering, Gifu University, Gifu 501-1112, Japan; alex@gifu-u.ac.jp; 4 TIER IV, Nagoya 450-6610, Japan

**Keywords:** world models, open-vocabulary semantics, generative models, BEV generation, continual learning, self-supervised learning, mobile robots, autonomous driving

## Abstract

Cognitive scientists believe that adaptable intelligent agents like humans perform spatial reasoning tasks by learned causal mental simulation. The problem of learning these simulations is called predictive world modeling. We present the first framework for a learning open-vocabulary predictive world model (OV-PWM) from sensor observations. The model is implemented through a hierarchical variational autoencoder (HVAE) capable of predicting diverse and accurate fully observed environments from accumulated partial observations. We show that the OV-PWM can model high-dimensional embedding maps of latent compositional embeddings representing sets of overlapping semantics inferable by sufficient similarity inference. The OV-PWM simplifies the prior two-stage closed-set PWM approach to the single-stage end-to-end learning method. CARLA simulator experiments show that the OV-PWM can learn compact latent representations and generate diverse and accurate worlds with fine details like road markings, achieving 69 mIoU over six query semantics on an urban evaluation sequence. We propose the OV-PWM as a versatile continual learning paradigm for providing spatio-semantic memory and learned internal simulation capabilities to future general-purpose mobile robots.

## 1. Introduction

Cognitive scientists believe that adaptable intelligent agents like humans represent the world internally using a small set of foundational cognitive components for perceiving inanimate objects, external agents, numeric concepts, social relations, and spatial environments [1]. These cognitive abilities allow intelligent agents to perform commonsense physical reasoning and imagine counterfactual scenarios to facilitate task accomplishment [2]. One of their key capabilities is predictive world modeling [3,4,5,6].

In contrast, mobile robots are conventionally designed and programmed for performing a priori specified tasks in known environments. General-purpose mobile robots on the other hand, aim to be flexible intelligent agents that can understand novel situations and complete a wide variety of tasks in new environments by leveraging world knowledge. Large language models (LLMs) have emerged as a promising direction for achieving general-purpose agents [7,8,9,10,11,12,13,14,15]. Core LLM agent abilities include understanding weakly specified goals defined in natural language [7], performing hierarchical planning through task decomposition [13,14,16,17,18] and program synthesis [12,13,14], and reasoning with commonsense world knowledge [15].

To complete a novel task in a new environment, a general-purpose mobile robot needs to comprehend the environment through an a priori unknown set of semantics. Vision language models (VLMs) [19,20,21,22,23,24,25,26,27,28,29] are a common approach to ground rich open-vocabulary (OV) semantics in the observed environment and connect the internal reasoning processes of LLM agents with the external world. However, spatio-semantic reasoning tasks may require information beyond what is currently observed. Efficiently fetching an item out of view requires a spatio-semantic memory of where the item is located [30]. Inferring navigational patterns like road lanes may require predictive assumptions of the unobserved environments behind obstructions [31]. A spatio-semantic memory [32] or scene representations [33] allow an agent to query semantics from observational memory [34,35,36] to navigate [37] and plan by reasoning [15]. Common representations of spatio-semantics include 3D reconstructions [38], object-centric and topological maps [39], scene graphs [40], and top-down metric grid maps [6].

The representation of spatio-semantic environment states for general-purpose agents thus requires they have the following properties: an encoded open-ended vocabulary of semantic concepts, the ability to represent and allow the querying of overlapping semantics (e.g., a *couch* is also a piece of *furniture*), and the ability to store observations compactly.

Latent compositional semantics [41] satisfies the above requirements.

**Figure 1:**
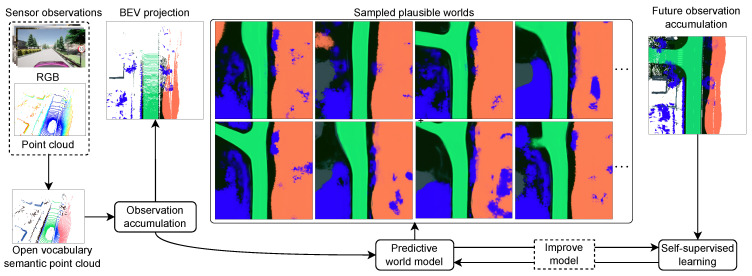
The framework integrates open-vocabulary semantic point cloud observations into a common vector space. A predictive world model samples a set of diverse plausible complete world states from a partially observed state. The model improves through continual learning from experience by comparing predicted and observed future states based on predictive coding. High-dimensional semantic embeddings are projected as RGB color values for visualization.

This work proposes an open-vocabulary predictive world model (OV-PWM) as a spatio-semantic memory and internal simulator for general-purpose mobile robots. The OV-PWM is a latent-variable generative model that learns from egocentric partial observations to predict complete environment states represented by grounded open-vocabulary semantics. The OV-PWM functions as an implementation of an artificial hippocampus that learns the distribution of compact latent codes, capturing the structure of observed environments. See Figure 1 for an overview of the model.

The explicit open-vocabulary environment representations enabled by OV-PWMs provide several potential advantages over implicit representations and conventional offline map-based mobile robots with human-annotated semantics. First, the OV-PWM can disambiguate an observed state by substituting unknown regions with plausible predictions based on prior observational experience. Committing to a particular complete state simplifies learning policies by removing the implicit marginalization of many plausible underlying states for state transition modeling. Secondly, OV-PWMs can integrate conventional map-based and perception-based planning and control methods. For example, safer motion planning may be achieved by sampling diverse plausible structures for unobserved regions and accounting for worst-case scenarios. Additional potential advantages include improving localization by densifying observations, verifying offline map consistency with the actually observed environment, and leveraging the highly expressive but compact latent state for planning in latent space [42]. Thirdly, learning a world model based on grounded open-vocabulary semantics allows us to optimize a single general OV-PWM for multiple tasks requiring different semantic perceptual information. Fourthly, leveraging unconditional open-vocabulary semantics supports the inferring of overlapping semantics via sufficient similarity inference [41].

The contributions of our paper are threefold:

- We propose an open-vocabulary predictive world model (OV-PWM) capable of predicting a diverse set of complete environment states represented by compositional latent semantic embeddings $h^{*}$ [41] by learning from observational experience only.
- We mathematically and empirically show that OV-PWMs can be learned end-to-end in a single stage, in contrast to prior conventional closed-set semantic PWMs with a two-stage optimization scheme [6].
- We empirically demonstrate that OV-PWMs can generate accurate and diverse plausible predictions in a new urban environment with fine semantic detail like *road markings*, reaching 69.19 mIoU on six query semantics.

We expand on our previous predictive world modeling conference paper [6] by extending our approach from probabilistic class semantics to open-vocabulary semantics, simplifying the learning method from a two-stage to a single-stage end-to-end paradigm and adding theoretical background including connecting the world model representation to the theory of latent compositional semantics [41], and provide experimental results demonstrating the accurate and diverse generation of high-dimensional embedding maps queryable by sufficient similarity semantic inference [41] using the CARLA (Car Learning to Act) simulator [43].

The rest of this paper is organized as follows: Section 2 explains how OV-PWMs connect several fields of artificial intelligence. In Section 3, we present how to translate observations into partial world states based on the theory of latent compositional semantics. In Section 4, we introduce the OV-PWM, including our training and inference methods. We present our experiments and results in Section 5 and Section 6 and summarize our findings in Section 7 and Section 8.

## 2. Background and Related Works

### 2.1. Arbitrary Conditional Density Estimation

The goal of arbitrary conditional density estimation [44,45,46] is to model the probability distributions $p(x_{u}|x_{o})$, where the random variables *x* are partitioned into observed $x_{o}$ and unobserved $x_{u}$ subsets. This partitioning can be interpreted differently based on the application domain. The methods for arbitrary conditional density estimation include various assumptions about how *x* is divided into $x_{o}$ and $x_{u}$. The primary objective is to generate diverse predictions while maximizing the likelihood of the observed data. In some applications, the partition of *x* into $x_{o}$ and $x_{u}$ can represent different states or transitions between states. The techniques for arbitrary conditional density estimation aim to model the conditional probability distribution $p(x_{u}|x_{o})$, where the observed variables $x_{o}$ serve as conditioning factors for predicting the unobserved variables $x_{u}$.

Image inpainting techniques aim to predict unobserved pixels $x_{u}$ from observed pixels $x_{o}$, which is analogous to the problem of predicting complete world states from partially observed states. A prevalent approach involves training an autoencoder (AE) [47] to compress partially observed images $x_{o}$ into compact latent codes *z*. These latent codes encode general visual patterns learned from reconstructing complete images *x* by leveraging common visual cues. The autoencoder is trained to reconstruct the complete image *x* from the partially observed input $x_{o}$, thereby enabling the prediction of unobserved pixels $x_{u}$.

Optimizing image inpainting models solely based on pixel-wise reconstruction can lead to a marginalization problem, where missing regions may be filled with multiple plausible pixel configurations, resulting in blurry outputs that represent the mean prediction when maximizing likelihood naively. To address this issue, several approaches have incorporated adversarial objectives. The Context Encoder [48] introduces an adversarial loss to improve the texture realism of inpainted regions. GLCIG [49] employs a coarse-to-fine generation scheme with diluted convolutions and two adversarial objectives: a global objective to ensure coherence across the entire image and a local objective to enhance detail. Yeh et al. [50] propose searching for the closest sample in an image database and using its latent code for prediction. Contextual Attention [51] incorporates an attention mechanism to facilitate long-distance information crossover and employs a two-stage coarse-to-fine generation process. Other works leverage application-specific biases for learning, such as facial semantic segmentation objectives [52]. In contrast, our framework demonstrates how hierarchical variational autoencoders (HVAEs) [53] and latent compositional semantics [41] enable the generation of structurally coherent environment representations with fine detail in a principled end-to-end manner, without requiring an adversarial objective.

Alternative approaches to image inpainting propose learning mask-aware convolutional filters. Liu et al. [54] introduce a special convolution filter and a mask update rule for propagating information about observed elements. Yu et al. [55] propose gated convolutions for learned mask updating. Our work demonstrates that open-vocabulary semantic embeddings naturally encode information about unobserved elements as zero vectors, distinct from the observed elements represented by unit vectors.

Pluralistic image inpainting focuses on stochastic state completion methods based on generative models. Generative adversarial network (GAN)-based methods [56,57] generate multiple plausible completions by conditioning on a random vector, often employing coarse-to-fine approaches. Variational autoencoder (VAE)-based methods [58] replace deterministic latent code with a sampling mechanism to allow for multiple plausible predictions. Previous works have improved training stability by constraining the latent distribution of partially observed images to match the distribution of fully observed images. PIC-Net [59] trains separate encoders for observable and unobservable image regions and matches the distributions between the two. UCTGAN [60] adds a cross-attention module to mix the latent representations of partially and fully observed images. DSI-VQVAE [61] applies VQVAE to stabilize training. Posterior matching [6,62] presents a method for the arbitrary conditioning of HVAEs by optimizing an additional partially observed sample encoder to match the latent distributions of a fully observed sample encoder. While posterior matching requires fully observed samples for training, our work experimentally demonstrates that HVAEs have the capacity to learn the high-fidelity generative models of not only images but also high-dimensional open-vocabulary embedding maps.

An alternative approach to predicting unobserved state variables from observed variables is to frame this as a missing data variational autoencoder (VAE) problem. This involves stochastic state completion, where the goal is to model the conditional probability distribution $p(x_{u}|x_{o})$. In low-dimensional settings, HI-VAE [63] derives a missing data evidence lower bound (ELBO) by removing contributions from unobserved data and replacing missing input data with zeros. EDDI [64] presents a partial VAE model that processes only observable elements by encoding them with positional encoding and using permutation-invariant operations similar to PointNet [65]. VAEM [66] is a two-layered hierarchical VAE (HVAE) for heterogeneous data which first transforms all input variables into a common latent space using type-specific encoders. HH-VAEM [67] demonstrates effective HVAE sampling using the Hamiltonian Monte Carlo algorithm. For high-dimensional data, Collier et al. [68] demonstrate the VAE effects of missing data on images. Our work extends prior missing-data VAE approaches by learning to model $p(x_{u}|x_{o})$ for high-dimensional representations without requiring fully observed ground truth samples for training. We leverage the capacity of HVAEs to learn high-fidelity generative models of not images but high-dimensional open-vocabulary embedding maps.

### 2.2. Bird’s-Eye View Generation

In mobile robotics, one approach to representing the environment is by generating top-down bird’s-eye view (BEV) maps from perception sensors, which serve as an alternative or complementary representation to human-annotated maps [31,69].

Camera-based methods have garnered significant attention due to the low cost of these sensors and their connection to biological vision systems. However, lifting 2D image observations to 3D representations is fundamentally an ill-posed problem. Inverse perspective mapping (IPM) [70,71,72] tackles this issue by assuming a flat ground plane, but this assumption often does not hold in real-world environments, leading to substantial projection errors. Approaches utilizing stereo cameras aim to address the lifting problem by inferring depth maps based on physical modeling principles. Nonetheless, the resulting depth maps tend to be noisy when mapping distant objects, object boundaries, and objects with indistinct textures. To overcome the limitations of stereo-based depth estimation, learning-based methods have been proposed. Cam2BEV [73] projects semantic features using IPM and refines the projection using a spatial transformer module trained on synthetic ground truth BEV data. Other approaches utilize learned monocular depth estimation [74,75,76,77,78,79] to lift 2D images to 3D point clouds, which are subsequently projected onto a top-down 2D grid to obtain bird’s-eye view (BEV) representations. Schulter et al. [80] introduce an adversarial objective that leverages ground truth maps to refine the predicted BEV representation. MonoLayout [81] learns its view transformation from self-supervised targets by integrating projected observations and using ground truth maps for BEV refinement. Later works propose probabilistic depth projection [82], categorical depth distribution networks [83], and multi-task learning [84] for BEV generation. VED [85] is a variational encoder trained on stereo vision data to predict low-resolution (64 × 64 px) semantic BEV representations from front-facing monocular images. Other methods employ multilayer perceptrons (MLPs) trained on ground truth maps [86,87,88] to lift images and generate BEV representations. SMERF [89] integrates coarse standard definition (SD) lane maps prior to using a transformer-based encoder to predict BEV lane maps from images.

Cross-attention-based transformer modules [90,91] and transformer architectures [92] have been employed to perform view transformations, lifting image features to bird’s-eye view (BEV) representations. Attention-based models tend to perform well at this task due to their global attention mechanism, which is not limited to processing neighboring pixel information like in convolutional neural networks (CNNs). However, attention-based models often require more data, effort, and computational resources for training and inference compared to CNNs, as they lack inductive biases. Our work differs from these view-transformation models in several ways. First, we leverage LiDAR data to achieve substantial improvements in projection and observation integration accuracy compared to image-only depth estimation methods. Second, our generative model can predict diverse plausible environment structures for unobserved regions, unlike view-transformation models, which typically are deterministic one-to-one functions. The ability to sample diverse predictions is crucial, as unobserved regions generally cannot be known deterministically [31].

While LiDAR-based bird’s-eye view (BEV) generation methods benefit from leveraging explicitly measured accurate distances for environment representation, some consider them prohibitively expensive for widespread deployment in mobile robots. Notable LiDAR-driven approaches include Fishing Net [88], which incorporates LiDAR information to enhance the spatial accuracy of BEVs generated through sensor fusion. MP3 [93] employs a learned module to generate map elements from LiDAR observations and ground truth map supervision, while HDMapNet [94] additionally incorporates image data. In contrast to these methods, our predictive world model framework does not rely on pre-existing ground truth maps for supervised training and can be trained solely on observational experience. Moreover, our method is generative and can provide diverse predictions, which is fundamentally necessary, as the correct prediction of occluded regions is generally indeterminable and there may be multiple plausible solutions.

### 2.3. World Models

The idea of using machine learning to learn predictive models of the world was proposed by Schmidhuber [3,4,5]. One prevalent approach leverages VAEs to extract latent state representations from perspective images [95,96]. These latent codes then serve as compressed world-state representations for planning actions. Recent advancements have incorporated adversarial learning to refine these latent codes [97,98] or employed contrastive learning with latent variables to model probabilistic transition dynamics [42].

An alternative research direction centers on inferring discrete object encodings from images. This approach draws inspiration from the concept of compositionality in human cognition [1]. Watters et al. [99] exemplify this strategy, employing a variational encoder to infer a set of latent object encoding vectors from a sequence of images, essentially utilizing a CNN-based module for this purpose. Building on the foundation of VAEs, subsequent research has focused on extracting more semantically rich object embeddings by leveraging the inherent neighborhood similarity arising from sampling stochasticity [100,101]. A noteworthy example is MONet [102], which utilizes a recurrent attention module to learn a variable number of semantic object encodings from its input. Recent extensions of MONet have highlighted the advantages of explicitly discovering objects for tasks involving future state prediction through compositional reasoning [103]. This approach extracts object encodings and learns relationships between them, facilitating predictions of future states using a GNN optimized with a contrastive loss function. Similarly, leveraging MONet as a foundation, works like COBRA [104] and DreamerV2 [105] have demonstrated their superior performance in reinforcement learning settings compared to state-of-the-art model-free methods [106,107].

Our work presents a distinct perspective on world modeling. We propose a method that learns an explicit, ego-agnostic 2D spatio-semantic representation of the environment’s state based solely on partial, agent-centric observations. This approach prioritizes interpretability and focuses on learning a world model from partial observations only. We believe that this method bridges the gap between leading world modeling approaches for game environments and real-world mobile robotics applications, where robots operate under conditions of partial observability.

### 2.4. Spatial AI

The conventional approach to building 3D environment representations in mobile robotics is simultaneous localization and mapping (SLAM) [108,109,110]. The core operation of SLAM involves calculating the optimal translation and rotation transformations to align successive point clouds. This allows for the creation of a unified map by accumulating aligned point clouds within a common reference frame. Loop closure optimization, which identifies and reconnects previously visited locations, is another crucial component of SLAM. Beyond geometric information, Semantic SLAM extends this framework to incorporate a semantic understanding of the environment or objects [111]. It can include estimates of object categories or segmentation information [112]. Our proposed framework leverages a similar principle of sensor observation integration, with the key addition of a predictive component that builds upon the strengths of established SLAM approaches.

Recent advancements in semantic mapping have moved beyond pre-defined semantic classes for specific tasks, venturing into the field of open-set semantics for general-purpose robotics applications. This shift utilizes open-vocabulary spatial representations that encode spatio-semantic maps using vision and language (VL) embeddings. These VL embeddings are typically generated by pre-trained models such as global VLMs [36], open-vocabulary object detectors [113], or dense VLMs [34,35,37]. Notably, the open-vocabulary approach allows for the querying of any semantic concept embedded within VL representations by leveraging cosine similarity with a query text embedding. As an alternative approach, neural radiance fields (NeRFs) [114] have emerged as a method for representing 3D objects [115,116] and environments [117,118] using neural networks. Recent work has extended NeRFs to capture open-vocabulary semantics [119]. The integration of large language models (LLMs) presents promising possibilities for spatio-semantic reasoning. This integration mimics the human vision-for-perception system through a top-down perceptual feedback loop [113,120], drawing inspiration from established models of human visual perception [121,122,123]. Our work contributes by demonstrating the capability of generative predictive world models to learn and predict high-dimensional, open-vocabulary semantic embeddings with high accuracy and diversity.

### 2.5. Open-Vocabulary Semantic Segmentation

Open-vocabulary semantic segmentation is a computer vision task that leverages the power of VLMs [19]. VLMs operate within a unified embedding space, enabling them to bridge the gap between visual and textual information. The core functionality of a global-description VLM involves training a visual encoder $Enc_{V}\left(\right)$ and a language encoder $Enc_{L}\left(\right)$ in tandem. These encoders operate on a paired image *x* and text description *t* to generate semantically aligned visual embeddings $z_{v}$ and textual embeddings $z_{t}$ within a shared embedding space Z. This alignment allows VLMs to act as an interface for querying visual data using natural language. Cosine similarity is typically used to measure the semantic similarity between $z_{v}$ and $z_{t}$. The training of these models often utilizes large-scale image-captioning datasets and contrastive learning techniques. While global description models hold promise for various applications, including image–text matching, multimodal searches, and visual question answering (VQA) [124,125], their outputs lack spatial grounding within the input image. This limitation hinders their effectiveness in tasks that require precise spatial reasoning, such as navigation, manipulation, and environment mapping [7,8,37].

In contrast, dense vision–language models [21,22,23,24,25,26,27,28,29,41] produce aligned embedding maps. These embedding maps represent semantic information at the pixel level, allowing for a more precise fit to object boundaries within the image. One approach to achieving densification involves modifying pre-existing global description models. Techniques like removing the final global pooling layer, as employed in MaskCLIP [25], leverage the strong generalization capabilities of these models. While this approach offers the benefit of utilizing pre-trained global description models, the resulting outputs often exhibit significant noise levels. This noise can significantly hinder the practical application of such models in real-world robotics tasks requiring accurate segmentation information.

An alternative approach to achieving dense descriptions leverages pre-trained region proposal (RP) models [126]. These models predict a set of object-masked bounding boxes. Each bounding box is then fed into a pre-trained global VLM [23] to generate a semantic embedding. This embedding is subsequently projected onto all pixels encompassed by the corresponding masked region within the original image. While the object-crop approach demonstrates promising results for the object-centric image inputs typical of small, controlled environments like kitchens or indoor spaces [29,127], it exhibits limitations in handling large-scale and complex scenes. Road environments, for instance, require multi-scale object perception, which this approach struggles to achieve effectively. Furthermore, the computational cost associated with performing individual inferences for each object can be significant.

In contrast to the previously discussed approaches, another research direction focuses on training a novel vision model specifically designed for dense feature representation. This model, denoted as $f_{\theta }\left(\right)$, leverages an architecture and optimization scheme tailored to this task. One example of such an approach is LERF [128]. LERF integrates language embeddings within a NeRF [114], enabling the semantic querying of 3D environment representations. This approach offers the potential for querying the environment based on semantic concepts. However, limitations exist. LERF may struggle with extrapolation tasks and potentially needs to observe the entire environment before functioning effectively. Open-vocabulary object detectors bridge the gap between semantic understanding and image regions by localizing predicted vision–language model (VLM) embeddings to bounding boxes [129]. Within the field of open-vocabulary semantic segmentation, two primary categories of models emerge: conditional and unconditional. Conditional models [24,28,130,131] facilitate fine-grained semantic segmentation guided by additional text or image inputs during the forward pass. However, this approach has limitations in projecting general-purpose, open-set semantics into a broader representation that encompasses both the spatial and semantic information of the environment. In contrast, unconditional methods [21,22,27,41] focus on predicting general-purpose embedding maps, enabling open-ended semantic querying after their projection. Notably, unlike global embedding models [19], unconditional open-vocabulary semantic segmentation models require smaller datasets with dense annotations for their training. The theory of latent compositional semantics [41] provides a valuable mathematical framework for understanding the representations learned by these unconditional models. This theory sheds light on the properties, guarantees, and representational capacity of these models. Our open-vocabulary predictive world model (OV-PWM) framework leverages open-vocabulary semantic segmentation to achieve accurate semantic projection onto environment states. This projection is facilitated by the theory of latent compositional semantics [41]. This theory provides valuable insights into the mathematical properties and representational capacity of the modeled semantic embeddings.

## 3. Open-Vocabulary Partial World States

This section describes how to generate open-vocabulary partial environment states from multimodal sensor observations. We leverage recent advances in unconditional open-vocabulary semantic segmentation based on the theory of latent compositional semantics [41] as our semantic representation. These partial world state representations serve as the input representations for learning the open-vocabulary predictive world models (OV-PWMs) described in Section 4.

### 3.1. Sensor Observation Processing

Mobile robot perception systems typically fuse complementary sensor modalities. Passively sensing RGB cameras provide rich semantic information. Actively sensing LiDARs or depth sensors provide accurate metric spatial perception. Sensor fusion approaches aim to leverage the complementary strengths of both vision modalities [88].

**Figure 2:**
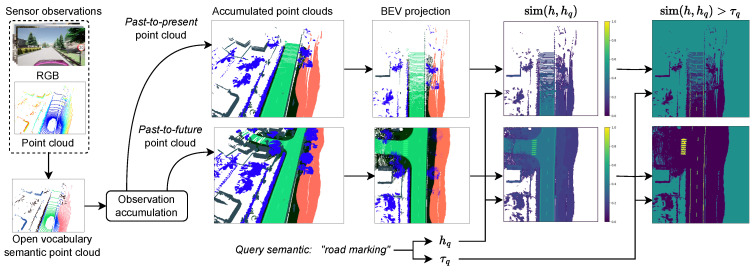
The process of transforming sensor observations into open-vocabulary partial world states. A semantic segmentation model interprets images. The inferred semantic embedding map is attached to a point cloud. Sequential semantic point clouds are accumulated into an ego-centric reference frame. Top-down projection creates BEV representations. BEVs can be measured for their similarity and sufficient similarity with a query semantic. High-dimensional semantic embeddings are projected as RGB color values for visualization.

Semantic point clouds are the natural unified data structure for representing both spatial and semantic information. A semantic point cloud is created by grounding semantic embedding maps extracted from 2D image pixels in spatial coordinates. The grounding is performed as follows: First, a point cloud is projected onto an image frame by a transformation specified by camera calibration parameters. Predicted open-vocabulary semantic embeddings are mapped to all points coinciding with the respective image’s coordinates. All points outside the image frame are discarded. The remaining set of points thus contain spatial information in the form of $\left(x,y,z\right)\in R^{3}$ coordinates and a semantic embedding $z\in R^{D}$ with dimensionality *D*, resulting in a semantic point cloud $P\in R^{N\times 3+D}$, where *N* is the number of semantically annotated points. See Figure 2 for visualized high-dimensional open-vocabulary semantic point clouds projected onto RGB values.

### 3.2. Open-Vocabulary Semantics

We propose mapping unconditional open-vocabulary, or latent compositional semantic embeddings, to point clouds. Here follows a brief explanation starting from conventional class semantics. A set of *K* class semantic embeddings are defined by separate basis vectors $e_{k}$ in an $R^{K}$ dimensional embedding space. Each semantic represented by $e_{k}$ is orthogonal to every other semantic $e_{l\neq k}$, meaning that every semantic is equally similar or dissimilar to every other semantic. Conventional class semantics therefore do not encode semantic similarity.

Open-vocabulary semantics instead has a fixed embedding space that is spanned by *D* orthogonal basis vectors $e_{1}\ldots e_{D}$ representing primitive latent semantics. All vectors $e_{d}$ define a latent prototypical semantic. All vectors in the embedding space are normalized and thus lie on the unit hypersphere $S^{D-1}$. A projection function $f_{\theta }\left(\right)$ maps any visual or text semantic *h* onto $S^{D-1}$. As all *h* are generally distributed over all basis vectors, the cosine similarity of two normalized embeddings

(1) $$\mathrm{sim}\left(h_{1},h_{2}\right)=\frac{h_{1}\cdot h_{2}}{\left|\right|h_{1}\left|||\right|h_{2}\left|\right|}={\left(h_{1}\right)}^{T}h_{2}$$

measures the relative semantic similarity.

Our predictive world modeling approach is based on interpreting RGB images using an unconditional open-vocabulary semantic segmentation model [132]. The segmentation model outputs a dense embedding map $H=R^{H\times W\times D}$, representing open-vocabulary semantics with a one-to-one pixel correspondence. A mathematical theory of unconditional open-vocabulary semantics [41] explains how models learn to output latent compositional semantics $h^{*}$ representing discriminable sets of membership semantics $H=\{h_{1},\ldots h_{K}\}$ as a hyperspherical cap $S_{cap}^{D-1}$ defined by $h^{*}$ and a sufficient similarity threshold τ. To compute if an observation *i* in a semantic point cloud represents a query semantic (e.g., if a point is *road*), the cosine similarity between the latent compositional semantic $h_{i}^{*}$ mapped to point *i* and the embedded query semantic $h_{q}$ must be higher than the sufficient similarity threshold of the query semantic $\tau_{q}$ and thus, in $S_{cap}^{D-1}$,

(2) $$\mathrm{sim}\left(h^{*},h_{q}\right)>\tau_{q}\Rightarrow MemberOf\left(\mathrm{point}\;i,\mathrm{query}\;\mathrm{semantic}\right).$$

The predicate MemberOf() in (2) denotes that all points *i* with latent compositional semantics $h^{*}$ are members of the set of all objects possessing the query semantic. In other words, point *i* is the query semantic (e.g., *road*), possibly in addition to other semantics (e.g., *road marking* and *drivable*). Set notation naturally allows for expressing that an object has membership with more than one semantic.

Conventional “most similar” open-vocabulary inference approaches [23] forgo knowing a sufficient similarity threshold τ and thus seemingly allow the querying of any never-encountered semantic. Nevertheless, the “most similar” inference approach has two fundamental flaws [21,41]: First, every point *i* can be a member of only one of the queried semantics. For example, a point on window-on-a-building-facade should be simultaneously inferable as both “window” and as part of a “building” at a higher level. Naively hard coding rules such as stating that “window” is also “building” are not generally true. Secondly, the set of query semantics is presumed to constitute a complete partitioning of all points, as even unrelated points will be mapped to one of the query semantics. For example, a *dog* queried by “grass” and “toy” is interpreted as “toy”. Naively using abstract word semantics like “other” as a substitute for unspecified semantics is not a principled solution as the similarity between the predicted semantic *h* and the unrelated query semantic $h_{q}$ is not guaranteed to be lower than the ambiguous meaning of “other”

(3) $$\mathrm{sim}\left(h,h_{other}\right)\overset{?}{\geq }\mathrm{sim}\left(h,h_{q}\right).$$

Sufficient similarity inferences are a principled solution to the flaws of “most similiar” inferences by allowing overlapping semantic inference (e.g., semantic membership with “window” and “building” can be simultaneously inferred) and inferring only true semantics, irrespective of the set of query semantics (e.g., *dog* is neither “grass” nor “toy”). In this work, we follow the theory of latent compositional semantics’ interpretation of unconditional semantics [41] and demonstrate the application of the sufficient similarity inference method for OV-PWMs.

In this work, we investigate whether or not high-dimensional open-vocabulary embeddings can be modeled using the predictive world model approach. We therefore do not consider the perception problem of inferring unconditional open-vocabulary semantics from images and instead leverage point clouds annotated with CARLA ground truth semantics [43] for experiments. We design a taxonomy in which each ground truth semantic is provided two additional high-level semantics (ex: a “road” is also a “drivable” and a “static” object). A single optimal latent compositional semantic embedding $h^{*}$ is computed as the mean centroid of the three associated semantics [41] and appended to each point to form an open-vocabulary semantic point cloud. We refer to prior work for in-depth investigations concerning learning and inferring open-vocabulary semantic embeddings from visual data [21,23,41,132]. The semantic taxonomy based on CARLA semantics is provided in Appendix A.

### 3.3. Observation Accumulation

The agent accumulates a sequence of unfiltered semantic point clouds $P^{\left(1\right)},\ldots ,P^{\left(T\right)}$ centered within the agent’s reference frame; over time t=1…T into a single semantic point cloud ${\bar{P}}^{\left(T\right)}$. This task is called point cloud registration or a scan matching problem [133]. We use the Iterative Closest Point (ICP) algorithm [134] to estimate the sensor’s motion and align sequential observations within the same reference frame. ICP takes the previous and latest point cloud and computes the transformation matrix $T_{t\to t+1}$ which best aligns with the previous point cloud $P^{\left(t\right)}$ to the latest one $P^{\left(t+1\right)}$. The matrix $T_{t\to t+1}$ corresponds to the agent’s motion between the two observations, as shown in (4). Multiplying the accumulated point cloud ${\bar{P}}^{\left(t\right)}$ with $T_{t\to t+1}$, as in (5), transforms all points into ${\tilde{P}}^{\left(t+1\right)}$ in the reference frame of the newest observations. This step is performed recursively every time step as new observations are perceived. Finally, we add the new observations $P^{\left(t+1\right)}$ to the transformed accumulated observations ${\tilde{P}}^{\left(t+1\right)}$, resulting in a new set of accumulated observations ${\bar{P}}^{\left(t+1\right)}$, as in (6)

(4) $$\begin{array}{cc} T_{t\to t+1} & =ICP(P^{\left(t\right)},P^{\left(t+1\right)}) \end{array}$$

(5) $$\begin{array}{cc} {\tilde{P}}^{\left(t+1\right)} & =T_{t\to t+1}{\bar{P}}^{\left(t\right)} \end{array}$$

(6) $$\begin{array}{cc} {\bar{P}}^{\left(t+1\right)} & =concatenate({\tilde{P}}^{\left(t+1\right)},P^{\left(t+1\right)}). \end{array}$$

A visual example of accumulated semantic point clouds is shown in Figure 2.

### 3.4. Partial World State Representation

The accumulated open-vocabulary semantic point cloud $\bar{P}$ encodes the agent’s observable environment into a sparse spatio-semantic 3D representation. However, conventional perception and planning methods benefit from a top-down 2D representation for computational efficiency. 2D discrete grids can be processed by the CNNs [135] and visual transformers (ViTs) [136] forming the backbone of state-of-the-art (SOTA) latent-variable generative models for images [53,137,138,139].

We generate the partial open-vocabulary semantic world state $x\in R^{H\times W\times D}$ by projecting $\bar{P}$ onto a 2D top-down bird’s-eye view (BEV) grid map spanning a region of size (H×W) around the agent. This projection method naturally handles non-flat surfaces such as sloping roads, as each 3D point’s (x,y,z) coordinates are projected onto a 2D point (x,y) with their (z) elevation coordinate subsumed. The subsumption of (z) can be understood visually by imagining viewing a perfectly flat and sloping straight road from above. From this perspective, both roads look geometrically equivalent, as would the projected BEV grid maps. Let (i,j) index a grid cell in *x*. For each point $p\in \bar{P}$ with coordinates (x,y,z), we compute the grid cell indices (i,j) and append $x_{i,j}$ with the semantic embedding *h* of *p*. The set of appended semantics $H=\{h^{\left(1\right)},\ldots ,h^{\left(K\right)}\}$ of all points *p* coinciding with the grid cell (i,j) are averaged into the centroid $h^{*}$ of H. The theory of latent compositional semantics provides mathematical guarantees of optimally retaining the original semantics of H [41]. A key advantage of open-vocabulary semantic embedding representations is their inherent discrimination of unobserved or unknown information using the zero vector $\vec{0}$. In contrast, observed information is represented by unit vectors *h* that lay on the hypersphere $S^{D-1}$. This naturally encodes ignorance into the model and enables it to distinguish unknown from empty regions during inference.

Leveraging the theory of latent compositional semantics with sufficient similarity inference [41] allows us to seamlessly represent and infer multiple overlapping semantics in the same grid cell (i,j). For example, a grid cell corresponding to a *road marking* may also possess *road* and *drivable* semantics, an inference which is not principally achievable by conventional “most similar” inferences, as explained in Section 3.2.

The presented open-vocabulary partial environment state *x* forms the input and learning signal for the open-vocabulary predictive world model described in the following section.

## 4. Open-Vocabulary Predictive World Model

Predictive world models (PWMs) aim to learn latent representations, capturing the underlying structure of the environment. PWMs, having learned this structure, are able to supplement their perception by predicting unobserved regions. Prediction generation follows the two-staged variational autoencoder (VAE) [58] latent variable approach: First, an encoder predicts a latent distribution p(z|x) of the objectively real world $x^{*}$, partially observed by sensors as *x*. Secondly, a particular latent variable *z* is sampled from p(z|x). Finally, a decoder maps *z* onto the most likely world $x^{*}$. This process is abstracted as the arbitrary conditioning latent-variable generative model $p(x^{*}|x)$. In this paper, we demonstrate how PWMs can learn $p(x^{*}|x)$ to sample diverse and plausible complete worlds $x^{*}$ from partially observed worlds *x* represented by open-vocabulary semantic embeddings $h\in R^{D}$ with dimension D>>1.

The primary challenge is to teach a generative model to predict complete worlds by predictive coding [140,141] from a set of partially observed incomplete worlds which are used as “ground truth” data only. In general, learning to predict “nothing” or “unknown” is easier than predicting plausible structures when lacking a complete ground truth learning signal to enforce commitment to a particular prediction. We employ the novel posterior matching latent-variable generative model as a solution which was introduced in our previous work [6]. In this work, we extend this approach to model high-dimensional open-vocabulary semantic embeddings and, in the process, simplify the previous two-stage approach into a single-stage end-to-end paradigm.

The open-vocabulary predictive world model (OV-PWM) is implemented by the SOTA hierarchical VAE (HVAE) model VDVAE [53], with an additional posterior matching encoder [6,62]. HVAEs [53,142,143] are capable of learning hierarchical latent variable distributions expressing a high degree of structure at different abstraction levels. HVAEs generalize autoregressive models [53], can achieve higher likelihoods than SOTA autoregressive models like PixelCNN [137] using fewer learned parameters, and generate samples orders of magnitude more quickly [53].

The following sections present a detailed description of the model and how it is trained and used for inference.

### 4.1. Latent Variable Generative Models

The goal of generative modeling is to approximate the distribution of p(x) by a learned model $p_{\theta }\left(x\right)$, maximizing the likelihood of the finite empirical dataset $D=\{x^{\left(1\right)},\ldots ,x^{\left(N\right)}\}$.

A latent-variable generative model p(x,z) approximates the joint distribution of observed variables or data *x* and compact latent variables or codes *z*. The problem can be factorized into a conditional model

(7) p(x,z)=p(x|z)p(z)

representing the process generating observed variables *x* from *z*, as well as the distribution of *z*. The problem is that learning $p_{\theta }\left(x\right)$ and $p_{\theta }\left(x|z\right)$ is computationally intractable for high-dimensional data when using naive methods due to the unknown interactive structure of *x* and *z*.

A solution is to reformulate the problem of learning $p_{\theta }\left(x\right)$ using approximate variational inference. Approximate variational inference proposes simultaneously learning an amortized inference function $q_{\theta }\left(z|x\right)$ and approximating the true latent representation distribution p(z|x) and the generative process $p_{\theta }\left(x|z\right)$.

The variational inference scheme used to optimize the likelihood of the generative model p(x) is derived as follows: The generative model p(x) is the marginal distribution of the joint distribution of the latent-variable generative model:

(8) $$p_{\theta }\left(x\right)=\int p_{\theta }\left(x,z\right)dz=\int p_{\theta }\left(z|x\right)p_{\theta }\left(x\right)dz=E_{z∼p_{\theta }\left(z|x\right)}p_{\theta }\left(x\right).$$

Taking the logarithm of both sides and leveraging the amortization factorization

(9) $$\begin{array}{cc} p_{\theta }\left(x,z\right) & =p_{\theta }\left(z|x\right)p_{\theta }\left(x\right) \end{array}$$

(10) $$\begin{array}{cc} p_{\theta }\left(x\right) & =\frac{p_{\theta }\left(x,z\right)}{p_{\theta }\left(z|x\right)} \end{array}$$

allows for a convenient decomposition

(11) $$\begin{array}{cc} \log p_{\theta }\left(x\right) & =E_{z∼p_{\theta }\left(z|x\right)}\log p_{\theta }\left(x\right) \end{array}$$

(12) $$\begin{array}{cc} \; & =E_{z∼p_{\theta }\left(z|x\right)}\log\frac{p_{\theta }\left(x,z\right)}{p_{\theta }\left(z|x\right)} \end{array}$$

(13) $$\begin{array}{cc} \; & =E_{z∼p_{\theta }\left(z|x\right)}\log\frac{p_{\theta }\left(x,z\right)q_{\phi }\left(z|x\right)}{p_{\theta }\left(z|x\right)q_{\phi }\left(z|x\right)} \end{array}$$

(14) $$\begin{array}{cc} \; & =E_{z∼p_{\theta }\left(z|x\right)}\log\frac{p_{\theta }\left(x,z\right)}{q_{\phi }\left(z|x\right)}+E_{z∼p_{\theta }\left(z|x\right)}\log\frac{q_{\phi }\left(z|x\right)}{p_{\theta }\left(z|x\right)} \end{array}$$

(15) $$\begin{array}{cc} \; & =\left[E\log p_{\theta }\left(x,z\right)-E\log q_{\phi }\left(z|x\right)\right]+\left[Eq_{\theta }\left(z|x\right)-Ep_{\theta }\left(z|x\right)\right]. \end{array}$$

The optimization objective is derived by denoting the first RHS term as $L_{\theta ,\phi }\left(x,z\right)$ and identifying the second RHS term as the KL divergence $D_{KL}\left(q_{\phi }\left(z|x\right),p_{\theta }\left(z|x\right)\right)$ and then rearranging these terms:

(16) $$\begin{array}{cc} \log p_{\theta }\left(x\right) & =L_{\theta ,\phi }\left(x,z\right)+D_{KL}\left(q_{\phi }\left(z|x\right),p_{\theta }\left(z|x\right)\right) \end{array}$$

(17) $$\begin{array}{cc} L_{\theta ,\phi }\left(x,z\right) & =\log p_{\theta }\left(x\right)-D_{KL}\left(q_{\phi }\left(z|x\right),p_{\theta }\left(z|x\right)\right). \end{array}$$

As $D_{KL}\left(q_{\phi }\left(z|x\right),p_{\theta }\left(z|x\right)\right)\geq 0$, it follows from (17) that

(18) $$L_{\theta ,\phi }\left(x,z\right)\leq \log p_{\theta }\left(x\right).$$

The optimization goal is to maximize $p_{\theta }\left(x\right)$, that is, the likelihood of data *x* according to the model $p_{\theta }\left(x\right)$. It follows from (17) that maximizing $L_{\theta ,\phi }\left(x,z\right)$ must necessarily maximize $p_{\theta }\left(x\right)$, as $L_{\theta ,\phi }\left(x,z\right)$ is a lower bound of $L_{\theta ,\phi }\left(x,z\right)$, making $L_{\theta ,\phi }\left(x,z\right)$ variational or the evidence lower bound (ELBO). The computable optimization objective for maximizing $L_{\theta ,\phi }\left(x,z\right)$ is derived by equivalently minimizing the negation of $L_{\theta ,\phi }\left(x,z\right)$:

(19) $$\begin{array}{cc} \max_{\theta ,\phi }L_{\theta ,\phi }\left(x,z\right) & =\min_{\theta ,\phi }-L_{\theta ,\phi }\left(x,z\right) \end{array}$$

(20) $$\begin{array}{cc} \; & =\min_{\theta ,\phi }-\left[E\log p_{\theta }\left(x,z\right)-Eq_{\phi }\left(z|x\right)\right] \end{array}$$

(21) $$\begin{array}{cc} \; & =\min_{\theta ,\phi }-\left[E\log p_{\theta }\left(x|z\right)-E\log p_{\theta }\left(z\right)-Eq_{\phi }\left(z|x\right)\right] \end{array}$$

(22) $$\begin{array}{cc} \; & =\min_{\theta ,\phi }-E\log p_{\theta }\left(x|z\right)+Eq_{\phi }\left(z|x\right)-E\log p_{\theta }\left(z\right) \end{array}$$

(23) $$\begin{array}{cc} \; & =\min_{\theta ,\phi }-E\log p_{\theta }\left(x|z\right)+D_{KL}\left(q_{\phi }\left(z|x\right),p_{\theta }\left(z\right)\right). \end{array}$$

The lower bound $L_{\theta ,\phi }\left(x,z\right)$, and, indirectly, the model likelihood $p_{\theta }\left(x\right)$, is therefore optimized by increasing $p_{\theta }\left(x|z\right)$ and decreasing $D_{KL}\left(q_{\phi }\left(z|x\right),p_{\theta }\left(z\right)\right)$.

The variational autoencoder (VAE) is a deep generative model that implements approximate variational inference. Both the amortized inference function $q_{\phi }\left(z|x\right)$ and generative model $p_{\theta }\left(x|z\right)$ are implemented by neural network function approximations. The VAE simultaneously learns $q_{\phi }\left(z|x\right)$ and $p_{\theta }\left(x|z\right)$ by inferring a distribution of the latent variable *z* and subsequently reconstructs the sampled *z* back into the observable variable *x*. The distribution of latent variables $p_{\theta }\left(z\right)$ is assumed to be a known distribution like the Normal distribution. The $D_{KL}\left(q_{\phi }\left(z|x\right),p_{\theta }\left(z\right)\right)$ term constrains the learned posterior distribution $q_{\phi }\left(z|x\right)$ to match the prior $p_{\theta }\left(z\right)$ so that new samples can be generated by simply sampling from the known distribution $p_{\theta }\left(z\right)$.

Vanilla VAEs suffer from constrained expressiveness due to being limited to a single set of latent variables *z*. This limitation is characterized by the generation of low-fidelity high-dimensional data like blurry high-resolution images.

The hierarchical VAE (HVAE) overcomes this limitation by introducing layers of latent variables $Z=(z^{\left(1\right)},\ldots ,z^{\left(K\right)})$. Each layer *k* models the structure of different levels of abstraction. The hierarchical order of latent variables naturally results in a decoupling of overall structure and visual appearance. The HVAE’s prior distribution, posterior distributions, and generative model can be factorized as

(24) $$\begin{array}{cc} p_{\theta }\left(Z\right) & =p_{\theta }\left(z_{1}|z_{2}\right)\ldots p_{\theta }\left(z_{K-1}|z_{K}\right)p_{\theta }\left(z_{K}\right) \end{array}$$

(25) $$\begin{array}{cc} q_{\phi }\left(Z|x\right) & =q_{\phi }\left(z_{1}|z_{2},x\right)\ldots q_{\phi }\left(z_{K-1}|z_{K},x\right)q_{\phi }\left(z_{K}|x\right) \end{array}$$

(26) $$\begin{array}{cc} p_{\theta }\left(x|Z\right) & =p_{\theta }\left(x|z_{1}\right)\ldots p_{\theta }\left(z_{K-1}|z_{K}\right)p_{\theta }\left(z_{K}\right) \end{array}$$

where all random variables *z* are modeled by Normal distributions N(z|μ,σ). Deeper or more abstract codes (i.e., $z_{K}$) encode the global structure, while shallow codes (i.e., $z_{1}$) encode the visual appearance of the elements in *x*. The deepest latent variable prior $p_{\theta }\left(z_{K}\right)$ is a known distribution like the Normal distribution in a VAE. However, subsequent priors $p_{\theta }\left(z_{K-1}\right)\ldots p_{\theta }\left(z_{1}\right)$ are learned priors for increased model expressivity.

### 4.2. Model Implementation and Training

**Figure 3:**
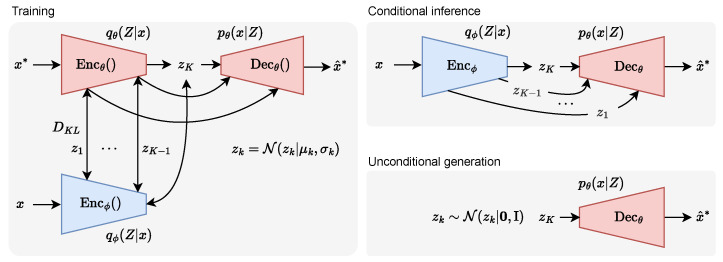
Predictive world model. The encoder ${\mathrm{Enc}}_{\theta }\left(\right)$ learns the hierarchical latent variables *Z* representing the environment ${\hat{x}}^{*}$ conditioned on the *past-to-future* partially observed state $x^{*}$. The posterior matching encoder ${\mathrm{Enc}}_{\phi }\left(\right)$ learns to predict the same distribution *Z* from the *past-to-present* state *x*. The decoder ${\mathrm{Dec}}_{\theta }$ learns to reconstruct diverse and plausible complete states ${\hat{x}}^{*}$ from *Z*.

We implement the OV-PWM based on the recent SOTA HVAE architecture called Very Deep VAE (VDVAE) [53]. This HVAE model has 48 layers of 16 dimensional latent variables (e.g., K=48) with incrementally increasing feature map resolution and decreasing intermediate feature dimensions throughout the layers. See Figure 3 for a visualization of training methodology explained in this section.

We use two inputs to train the model. The first input is the presently observed world $x\in R^{H\times W\times D}$ (e.g., *past-to-present* accumulated observations). The second input is the future observed world $x^{*}\in R^{H\times W\times D}$ (e.g., *past-to-future* accumulated observations). *x* and $x^{*}$ are high-dimensional grid maps with elements representing normalized open-vocabulary semantic embeddings $h\in S^{D-1}$ with dimension *D*. Unobserved elements are represented by the zero vector 0.

The two inputs are processed by two structurally identical but separate encoders. The future observed world $x^{*}$ is processed by the encoder $Enc_{\theta }\left(x^{*}\right)$, approximating $q_{\theta }\left(Z|x^{*}\right)$, into intermediate feature maps $Y^{*}=\left\{y_{1}^{*}\ldots y_{K-1}^{*}\right\}$ and the latent feature vector $y_{K}^{*}$. The presently observed world *x* is processed by the posterior matching encoder $Enc_{\phi }\left(x\right)$, approximating $q_{\phi }\left(Z|x\right)$ into $Y=\{y_{1}\ldots y_{K-1}\}$ and the latent feature vector $y_{K}$.

A single decoder generates a sample ${\hat{x}}^{*}$ by first sampling the latent variable $z_{K}$ from the distribution conditioned on $y^{*}K$. The intermediate reconstruction ${\tilde{x}}_{K}^{*}$ is computed from $z_{K}$ and learned bias variables. Subsequent latent variables zk are sampled by the corresponding intermediate feature maps $y_{k}^{*}$ from the encoder and the previous intermediate reconstruction ${\tilde{x}}_{k-1}^{*}$. Subsequent intermediate reconstructions ${\tilde{x}}_{k}^{*}$ are computed based on the sampled $z_{k}$ and ${\tilde{x}}_{k-1}^{*}$. The features $Y=\{y_{1}\ldots y_{K}\}$ outputted by the posterior matching encoder $Enc_{\phi }\left(x^{*}\right)$ are optimized to predict the same latent distribution $q_{\theta }\left(z_{k}\right)$ as the $q_{\theta }\left(z_{k}\right)$ distribution outputted by the future observation encoder $Enc_{\theta }\left(x^{*}\right)$.

The final intermediate feature map ${\tilde{x}}^{*}\in R^{H,W,D^{\prime }}$ is mapped onto an open-vocabulary semantic embedding map ${\hat{x}}^{*}\in R^{H,W,D}$ via a linear projection. Forcing the output to lie on the hypersphere $S^{D-1}$ and thus represent the latent compositional semantic denoting the set of the most likely membership semantics

(27) $$\forall x_{i,j}\in x∼p_{\theta }\left(x|Z\right)\Rightarrow x_{i,j}\in S^{D-1},$$

resolves the problematic tendency of the previous semantic probability approach [6]. The prior probabilistic closed-set semantics approach represents membership semantics as the *K* probability that element (i,j) is a member of the semantic k∈K. Forcing the model to predict a latent compositional semantic embedding *h* naturally allows for the inferring of overlapping semantics while overcoming the maximum likelihood shortcut learning problem of readily predicting “unknown” instead of penalizing committing to a misprediction. Uncertainty can instead be estimated by stochastic variation from repeatedly sampling the posterior [144]. Our prior two-stage approach with intermediate pseudo-ground truth states is not needed for OV-PWMs, thus simplifying the method to a single-stage end-to-end learning process.

The $Enc_{\theta }\left(\right)$ and $Dec_{\theta }\left(\right)$ components of the dual encoder HVAE are optimized by maximizing the hierarchical ELBO

(28) $$\max_{\theta ,\phi }L_{\theta ,\phi }\left(x,Z\right)=\min_{\theta ,\phi }E\left[-\log p_{\theta }\left(x|Z\right)+D_{KL}(q_{\phi }\left(Z|x\right)||p_{\theta }\left(Z\right))\right]$$

where $\log p_{\theta }\left(x|Z\right)$ is the likelihood of the sample $x^{*}$, reconstructed from *Z*, and a KL divergence term that measures the separation between the learned posterior and prior distributions.

(29) $$D_{KL}\left(q_{\theta }\left(Z|x\right)\left|\right|p_{\theta }\left(Z\right)\right)=\sum_{k=2}^{K}q_{\theta }\left(z_{\geq k}|x\right)\left[D_{KL}\left(q_{\theta }\left(z_{k-1}|z_{k},x\right)∥p_{\theta }\left(z_{k-1}|z_{k}\right)\right)\right]+D_{KL}\left(q_{\theta }\left(z_{K}|x\right)\left|\right|p_{\theta }\left(z_{K}\right)\right)$$

We simultaneously train the secondary posterior matching encoder $Enc_{\phi }\left(\right)$ to predict latent distributions *Z* for partially observed environments *x* which are similar to the *Z* inferred from the regular encoder $E_{\theta }\left(x^{*}\right)$ using future observed worlds $x^{*}$. The second posterior matching encoder is optimized by minimizing

(30) $$D_{KL}\left(q_{\phi }\left(Z|x^{*}\right)∥q_{\Psi }\left(Z|x_{po}\right)\right)=\sum_{k=1}^{K}q\left(z_{>k}|x\right)\left[D_{KL}\left(q_{\phi }\left(z_{k}|z_{>k},x^{*}\right)\left|\right|q_{\psi }\left(z_{k}|z_{>k},x_{po}\right)\right)\right].$$

Maximizing the likelihood of $p_{\theta }\left(x|Z\right)$ in (28) is equivalent to minimizing the cosine distance of normalized OV semantic embeddings modeled by the OV-PWM.

(31) $$\min E-\log\left(p|Z\right)=\min E\left(1-\mathrm{sim}\left(x,\hat{x}\right)\right)=\min E\left(1-x^{T}\hat{x}\right).$$

The practical formulation of the hierarchical ELBO (28) used for optimizing the OV-PWM is therefore

(32) $$\max_{\theta ,\phi }L_{\theta ,\phi }\left(x,Z\right)=\min_{\theta ,\phi }E\left[\left(1-x^{T}\hat{x}\right)+D_{KL}(q_{\phi }\left(Z|x\right)\left|\right|p_{\theta }\left(Z\right))\right].$$

See Appendix C for a derivation of (31).

### 4.3. Model Inference

At the time of inference the model uses the posterior matching encoder $Enc_{\phi }\left(\right)$ to generate a latent distribution *Z* that can be decoded by $Dec_{\theta }\left(\right)$ into a predicted complete world state ${\hat{x}}^{*}$. The model can be used for unconditional generation by incrementally sampling latent variables *Z* from the learned prior distribution $q_{\theta }\left(Z\right)$. The regular encoder $Enc_{\theta }\left(\right)$ trained on future observations $x^{*}$ is not used during inference. See Figure 3 for a visualization of the conditional and unconditional inference procedure.

## 5. Experiments

In this section, we describe the experiments conducted to measure how well an open-world predictive world model (OV-PWM) can learn a compact latent representation of environments represented by high-dimensional open-vocabulary embeddings.

We set up our experiments using the open source autonomous driving simulator CARLA [43]. This simulator provides a set of realistic 3D environments and a traffic manager and supports the accurate rendering of synchronized sensor data streams like RGB images, depth maps, and LiDAR point clouds. We used the latest 0.9.15 release. The reasons for using CARLA are two-fold: First, CARLA allows us to evaluate the predictive accuracy of fine semantics by providing ground truths for road markings. Common real-world datasets like SemanticKITTI [145] and KITTI-360 [146] do not provide road marking ground truths. Secondly, implementing our experiments in a simulator facilitates future work involving closed-loop autonomous driving research experiments leveraging OV-PWMs.

The experimental set up is explained next. We ran the simulator and collected approximately 20 min of observational experience from environments *Town05*, *Town06*, *Town07*, and *Town10* as observational experience, or training data. A separate environment *Town04* was used for evaluation. The environments were chosen based on the presence of road marking semantics. We computed and appended ideal latent compositional semantics to the point cloud according to a three level taxonomy with overlapping semantics, as explained in Section 3.2. The semantic taxonomy is provided in Appendix A. The semantics are encoded as 768-dimensional SBERT embeddings [147]. Next, we processed the sequential observations into accumulated semantic point clouds, as explained in Section 3.3. All points 2 m above the ground were filtered. Dynamic objects were filtered by sufficient similarity inference. From the accumulated point clouds we generatef BEV partial world representations as explained in Section 3.4. We used the same translation and warping data augmentation technique as detailed in prior work [6] on model training samples to improve their generalization. The evaluation samples are not augmented. The resulting number of training and evaluation samples were 7145 and 178 samples, respectively.

The HVAE model was trained on the generated training samples for 180K iterations for four days using six A6000 GPUs, as detailed in Section 4.2. See the public code repository for hyperparameter details. The trained HVAE model was evaluated on a separate evaluation set of unaugmented samples.

The following two metrics are employed to measure the accuracy of the OV-PWM model. First, semantic similarity between the predicted embedding maps ${\hat{x}}^{*}$ and future observed worlds $x^{*}$ is measured as the mean cosine distance between the predicted and observed open-vocabulary embeddings $x_{i,j}^{*}\in S^{D-1}$ and ${\hat{x}}_{i,j}^{*}\in S^{D-1}$ covered by the observed element mask *M*

(33) $$\mathrm{sim}\left(x^{*},{\hat{x}}^{*}\right)=\frac{1}{\left|M\right|}\sum_{(i,j)\in M}\mathrm{sim}(x_{i,j}^{*},{\hat{x}}_{i,j}^{*})=\frac{1}{\left|M\right|}\sum_{(i,j)\in M}{\left(x_{i,j}^{*}\right)}^{T}\cdot {\hat{x}}_{i,j}^{*}.$$

Secondly, semantic accuracy is measured by the intersection over union (IoU) of queried semantics. We compute the IoU based on a sufficient semantics interpretation of unconditional open-vocabulary semantics according to the theory of latent compositional semantics [41]. The OV embedding maps ${\hat{x}}^{*}$ and $x^{*}$ are first checked element-wise for their membership within the query semantic by an a priori computed sufficient similarity threshold value $\tau_{\mathrm{sem}}$

$$b_{i,j}=\left\{\begin{array}{cc} T, & \mathrm{if}\;\mathrm{sim}\left(x_{i,j}\right)>\tau_{sem}\\ F, & \mathrm{otherwise} \end{array}\right.$$

resulting in the boolean maps *b* and $\hat{b}$, with elements represented as true T and false F. The query semantic IoU is computed as

(34) $$\mathrm{IoU}\left(x^{*},{\hat{x}}^{*}\right)=\frac{\sum_{(i,j)\in M}\;b_{i,j}\cap {\hat{b}}_{i,j}}{\sum_{(i,j)\in M}\;b_{i,j}\cup {\hat{b}}_{i,j}}$$

with the boolean map $\hat{b}$ obtained from ${\hat{x}}^{*}$ considered as the ground truth target. The mean IoU (mIoU) is used to quantify the simulator’s performance over a set of query semantics *H*

(35) $$\mathrm{mIoU}=\frac{1}{H}\sum_{h\in H}{\mathrm{IoU}}_{h}.$$

We estimated the optimal sufficient similarity threshold values for query semantics $\tau_{q}$ using logistic regression models, maximizing likelihood over the trained split observations and following prior work [41]. The optimal $\tau_{q}$ is the decision boundary or $\left(sim\right)\left(x,x_{q}\right)$ separating true positive and negative points with least error according to the model

(36) $$\tau_{q}=\max\left[\mathrm{MemberOf}\left(x,q\right)\;p(\mathrm{MemberOf}\left(\mathrm{sim}\left(x,x_{q}\right)\geq \tau_{q},q\right))\right].$$

We provided a set of unconditionally sampled world states ${\hat{x}}^{*}$ to assess the robustness of the learned open-vocabulary world model. Unconditional generation starts by randomly sampling the deepest latent variable $z_{K}\in R^{16}$ in (26) and generates ${\hat{x}}^{*}$, without conditioning on the partially observed world *x*, as input.

## 6. Results

In this section, we present the results of the CARLA simulator experiment. The results show that environments represented by high-dimensional open-vocabulary semantic embeddings can be accurately modeled using the predictive world modeling approach. Additionally, we analyze the results from the perspective of potential real-world large-scale applications.

Table 1 shows the semantic IoU prediction accuracy for an urban environment sequence not in the training sample. We applied a “best of *N* samples” evaluation approach [6] to demonstrate how the sampling of diverse structures improves the likelihood of predicting the actual world from partial observations. The mean IoU prediction over all elements (i,j) and semantics is 65.13 with 1 sample and increases to 69.19 with 32 samples. Modeling and predicting fine spatial patterns like *road markings* is challenging and reaches only 22.99 IoU over 32 samples. The advantage of generative modeling is most apparent in less predictable large semantic structures like *vegetation* and *sidewalk*, as sampling increases their accuracy by 9.45 and 9.10 IoU points, respectively. Over all semantics, sampling increases the mean IoU by 4.06 IoU points.

Table 2 shows the IoU prediction accuracy of a highway sequence not in the training distribution. The model’s predictive performance in highway environments is generally higher than urban environments due to their higher determinism. However, *road marking*’s predictability is lower due to lacking localized contextual cues such as intersections and narrow road structures.

**Figure 4:**
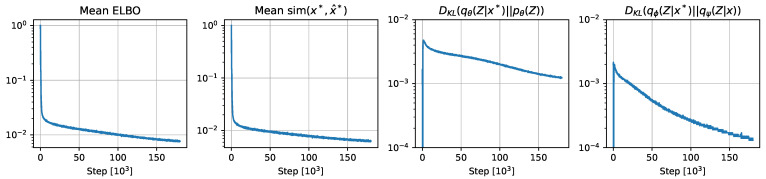
Training plots. The mean ELBO (28), cosine distance (31), posterior (29), and posterior matching (30) distribution separation metrics continue to decrease with additional computation. See Section 4.2 for an explanation of the partially observed states *x*, $x^{*}$, and predicted complete states ${\hat{x}}^{*}$.

Table 3 shows the model’s performance on a random subset of 200 samples from the training set. The results indicate that model training is not yet saturated on the limited training dataset, as semantics like *road marking*, *side walk*, and *vegetation* have room to improve. Comparison with the test set performance given in Table 1 shows comparable performance is achieved with the training set, meaning generalization is achieved. As the training performance continues to improve log linearly, as shown in Figure 4, it is reasonable to conclude that its generalization performance will continue to improve with additional training.

Our proposed OV-PWM framework lacks direct comparative baselines. To the best of our knowledge, only our prior work leverages LiDAR point clouds with generative modeling to predict spatial environments without requiring ground truth map data [6]. The prior closed-set predictive world model trained on KITTI-360 data [146] was quantitatively evaluated only for *road* semantics and achieved 98.73 IoU. We consider our open-vocabulary urban environment result of 94.33 to be of comparable quality and thus conclude that learning open-vocabulary world models perform equivalently to closed-set world models while greatly simplifying their learning method to a one-stage end-to-end paradigm, as explained in Section 3.4.

Other comparative baselines include image-based methods which generally are not used for generative models trained and evaluated on the same ground truth data domain (e.g., within the same city). One such baseline is a recent SOTA image-based monocular model [90] which achieved 68.34 road IoU on the KITTI Raw dataset [148]. Their performance differences exemplify the advantage of leveraging LiDAR point clouds, as our method does.

**Figure 5:**
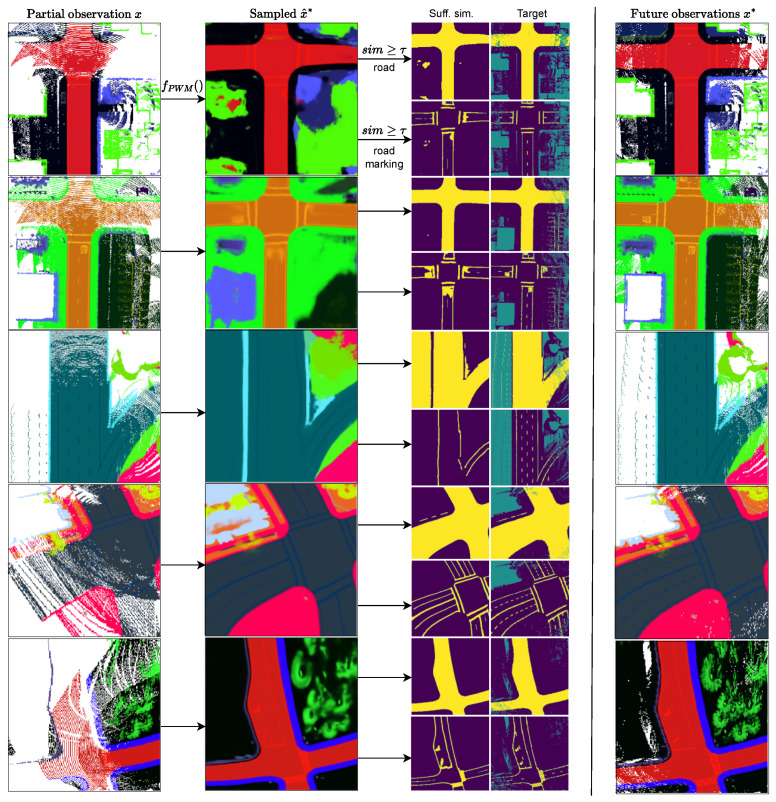
Conditional sampling visualizations. The high-dimensional open-vocabulary partial observation input *x* and sampled predictive world model output ${\hat{x}}^{*}$ are projected onto RGB images by PCA projection. Semantic inferences by sufficient similarity are shown in the third column. The actual worlds perceived in future observations are shown in the forth column. The first three rows show evaluation samples. The remaining two rows show samples from the training distribution.

Figure 5 provides visual examples of plausible world samples ${\hat{x}}^{*}$ generated from partial observations *x*. Examples of semantic inference by sufficient similarity are shown. The actual world perceived in future observations is included for comparison. The examples illustrate how large structures like *road* are accurately learned. Finer semantic details like *road markings* are comparatively challenging to represent and predict. However, the training samples display an improved granularity of their fine semantics, indicating that further training on a larger training set covering additional patterns may enhance their performance.

**Figure 6:**
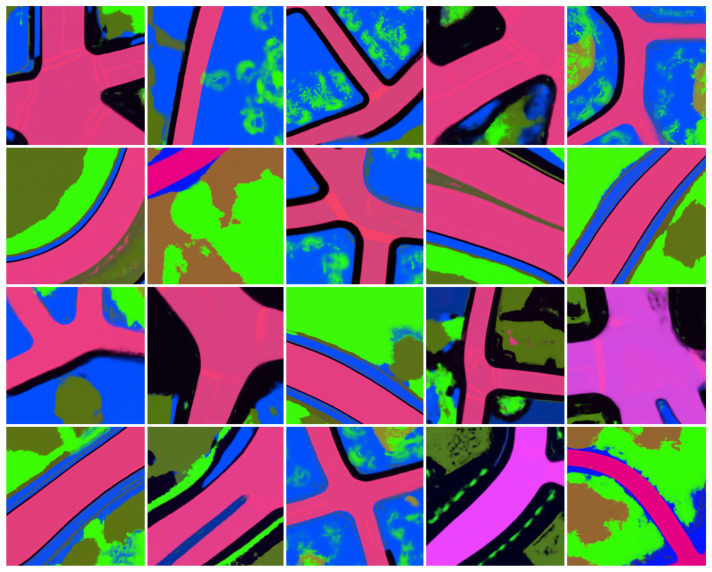
Unconditional sampling visualizations. High-dimensional open-vocabulary embedding maps are generated by the predictive world model $p_{\theta }\left(x|Z\right)$ through sampling from the learned prior distribution $p_{\theta }\left(Z\right)$. The embedding maps are visualized as RGB images by PCA projection.

Figure 6 displays a set of randomly sampled environments from the learned prior distribution $p_{\theta }\left(Z\right)$. The sampled environments showcase intricate details like road markings and semantically plausible configurations. Some generated samples are partially degenerate. Additional optimization of the learned prior $p_{\theta }\left(Z\right)$ and generative model $p_{\theta }\left(x|Z\right)$ is expected to reduce the likelihood of degenerate samples. Figure 4 shows that both $p_{\theta }\left(Z\right)$ and $p_{\theta }\left(x|Z\right)$ are likely to improve with additional training.

The predictive world model’s mean inference time is 0.175 s or 5.71 Hz on an RTX 4090 GPU. Our method is thus applicable for real-time applications, given a modern SLAM implementation [111,149,150] is capable of operating faster than sensor frame rates.

## 7. Discussion

A limitation of our current approach is its top-down 2D grid representation. Two-dimensional embedding maps do not represent vertical information or multi-layered environments, which are required for general 3D representations. Extending the OV-PWM approach to 3D representations using voxel grids or neural radiance fields is a promising direction for future work and will enable spatial reasoning in fully general complex 3D structures. While the model already demonstrates a promising generalization capability in new environments, the modeling of finely detailed semantics like *road markings* displays room for improvement. Given that the original VDVAE model was trained on 32 V100 GPUs for 2.5 weeks (*we: 6 A6000 GPUs for 4 days*) on a large dataset of 70,000 samples [53] (*we: 7000 samples*), and the OV-PWM’s training performance trend indicates room for further improvement, it is reasonable to expect additional training time and diverse observational experiences to further boost performance. Reducing degenerate samples resulting from inaccurate and erroneous ICP scan matching steps by implementing a robust SLAM-based observation accumulation framework may further improve training efficiency. Despite limited computational resources, the training set’s size, and degenerate samples, our method learns to generate outputs with intricate details emerging even from unconditional sampling based on prior distributions. Other directions include incorporating agents and temporal dynamics into predictive world models, as well as demonstrating the advantages of learned simulators in practical embodied task planning and decision making problems, using large-scale real-world data.

## 8. Conclusions

The experimental results validate our research hypothesis, suggesting that the open-vocabulary predictive world model (OV-PWM) can learn to predict accurate and diverse fully observed environment representations encoded by high-dimensional latent compositional semantic embeddings [41] from partial egocentric observations only. By capturing this structure in a compact latent code in an easily sampled learned prior distribution, the OV-PWM model can “imagine” and predict the unobserved regions of an environment given a partial view of it. The OV-PWM model achieves predictive accuracy and diversity comparable to our previous probabilistic closed-set predictive world model [6] with the advantage of supporting open vocabulary and overlapping semantic inferences, as required for future general-purpose mobile robots. Our framework also simplifies its learning method to a single-stage end-to-end paradigm, whereas the previous approached required a two-stage optimization scheme.

Overall, we propose OV-PWMs as a promising direction for endowing general-purpose mobile robotic agents with spatio-semantic environment representations and an internal simulator. The OV-PWM allows an agent to imagine the possible configurations of unmapped regions by learning an explicit generative model of environments represented by open-vocabulary semantics, potentially facilitating safer planning, continual mapping, and spatio-semantic reasoning. The ability to distill vast observational experience into a set of compact latent representations brings us closer to replicating the key cognitive abilities of biological intelligence.

## Figures and Tables

**Table 1:** World model prediction accuracy when using “best of *N* samples” on the urban test sequence.

		IoU					
#Samples		1	2	4	8	16	32
road	All	92.75	93.36	93.61	93.89	94.20	94.33
	Unobs.	84.07	85.70	86.10	86.69	87.54	87.74
road marking	All	21.02	21.21	21.95	22.31	22.91	22.99
	Unobs.	12.85	13.77	14.24	14.84	15.47	16.00
side walk	All	51.39	53.45	56.49	57.38	59.57	60.49
	Unobs.	41.50	45.51	48.72	50.33	52.07	52.53
vegetation	All	34.91	37.25	40.54	41.67	43.42	44.36
	Unobs.	28.11	31.97	35.08	36.27	37.96	40.02
static	All	97.61	97.61	97.85	98.08	98.12	98.23
	Unobs.	97.73	97.88	98.15	98.22	98.35	98.40
drivable	All	93.10	93.69	93.94	94.25	94.63	94.71
	Unobs.	84.89	86.60	87.00	87.55	88.52	88.72
mIoU	All	65.13	66.10	67.40	67.93	68.81	69.19
	Unobs.	58.19	60.24	61.55	62.32	63.32	63.90

**Table 2:** World model prediction accuracy when using “best of *N* samples” on the highway test sequence.

		IoU					
#Samples		1	2	4	8	16	32
road	All	98.01	98.15	98.20	98.29	98.31	98.34
	Unobs.	95.93	96.68	96.96	97.14	97.28	97.44
road marking	All	9.90	11.15	11.19	12.02	12.19	13.20
	Unobs.	9.51	10.61	10.67	11.53	12.09	12.49
vegetation	All	38.29	38.64	39.22	40.01	40.23	40.45
	Unobs.	43.11	43.68	44.27	44.83	45.37	45.33
static	All	98.54	98.73	98.79	98.83	98.88	98.90
	Unobs.	95.10	96.40	96.64	97.10	97.36	97.49
drivable	All	98.02	98.15	98.21	98.29	98.31	98.34
	Unobs.	95.91	96.62	96.92	97.07	97.23	97.41
mIoU	All	68.55	68.96	69.12	69.49	69.58	69.85
	Unobs.	67.91	68.80	69.09	69.53	69.87	70.03

**Table 3:** World model prediction accuracy when using “best of *N* samples” on the training set.

		IoU					
#Samples		1	2	4	8	16	32
road	All	97.16	97.20	97.30	97.35	97.38	97.42
	Unobs.	95.00	95.27	95.45	95.63	95.79	95.89
road marking	Obs.	34.14	34.33	34.62	34.83	35.04	35.24
	Unobs.	26.79	27.06	27.57	28.13	28.37	28.53
side walk	All	58.23	58.36	58.56	58.93	59.10	59.11
	Unobs.	55.03	56.17	56.20	57.12	57.54	57.69
vegetation	All	75.44	76.06	76.71	76.98	77.21	77.57
	Unobs.	66.33	68.01	68.67	70.03	71.29	71.89
static	All	98.79	98.81	98.81	98.82	98.83	98.84
	Unobs.	98.47	98.56	98.56	98.59	98.61	98.62
drivable	All	97.27	97.32	97.41	97.47	97.51	97.55
	Unobs.	95.47	95.83	96.09	96.25	96.27	96.39
mIoU	All	76.84	77.01	77.24	77.40	77.51	77.62
	Unobs.	72.85	73.48	73.76	74.29	74.65	74.84

## Data Availability

All data used for experimental evaluations were generated by the CARLA simulator 0.9.15 release. The data, model, and code used for these evaluations will be uploaded to a public repository for reproducibility upon their acceptance.

## Appendix A. Semantic Taxonomy

**Table A1:** Semantic taxonomy used for the CARLA simulator experiments.

Level 1 (CARLA)	Level 2	Level 3
unlabeled	unlabeled	unlabeled
road	drivable	static
side walk	ground	static
building	construction	static
wall	construction	static
fence	structural	static
pole	structural	static
traffic light	traffic information	static
traffic sign	traffic information	static
vegetation	plant	static
terrain	ground	static
sky	sky	sky
pedestrian	person	dynamic
rider	person	dynamic
car	vehicle	dynamic
truck	vehicle	dynamic
bus	vehicle	dynamic
train	vehicle	dynamic
motorcycle	vehicle	dynamic
bicycle	vehicle	dynamic
static	static	static
dynamic	dynamic	dynamic
other	other	other
water	fluid	dynamic
road marking	road	drivable
ground	static	static
bridge	construction	static
rail track	metal	static
guard rail	structural	static

## Appendix B. Sufficient Similarity Threshold Values

**Table A2:** Sufficient similarity threshold values used for filtering and semantic inferences.

Semantic	Suff. Sim. τ
road	0.5934
road marking	0.3944
side walk	0.481
vegetation	0.4872
static	0.456
drivable	0.5429
dynamic	0.6300

## Appendix C. Deriving Cosine Distance from Negative Log Likelihood Minimization

Here we show that minimizing the negative log likelihood p(x|Z) in (28) is equivalent to minimizing the cosine distance of normalized OV semantic embeddings modeled by the OV-PWM. By proposing that the output variable distribution is a Normal distribution and presuming the stochastic process variance $\sigma^{2}$ is constant and thus does not affect the minimization objective,

(A1) $$\begin{array}{cc} \min-\log(p(x|Z) & =\min-\log N(x|\mu \left(Z\right),2\sigma^{2}I) \end{array}$$

(A2) $$\begin{array}{cc} \; & =\min-\log\left[\frac{1}{\sqrt{2\pi \sigma^{2}}}\exp\left(-\frac{1}{2}\frac{{\left(x-\mu \left(Z\right)\right)}^{2}}{\sigma^{2}}\right)\right] \end{array}$$

(A3) $$\begin{array}{cc} \; & =\min-\left[\log{\left(2\pi \sigma^{2}\right)}^{-\frac{1}{2}}-\frac{1}{2}\frac{{\left(x-\mu \left(Z\right)\right)}^{2}}{\sigma^{2}}\right] \end{array}$$

(A4) $$\begin{array}{cc} \; & =\min\frac{1}{2}\left[\log\left(2\pi \sigma^{2}\right)+\frac{{\left(x-\mu \left(Z\right)\right)}^{2}}{\sigma^{2}}\right] \end{array}$$

(A5) $$\begin{array}{cc} \; & \propto \min\frac{1}{2}{\left(x-\mu \left(Z\right)\right)}^{2} \end{array}$$

(A6) $$\begin{array}{cc} \; & =\min\frac{1}{2}{\left(x-\mu \left(Z\right)\right)}^{T}\left(x-\mu \left(Z\right)\right) \end{array}$$

(A7) $$\begin{array}{cc} \; & =\min\frac{1}{2}\left(x^{T}x-2x^{T}\mu \left(Z\right)+\mu {\left(Z\right)}^{T}\mu \left(Z\right)\right) \end{array}$$

(A8) $$\begin{array}{cc} \; & =\min\frac{1}{2}\left(1-2x^{T}\mu \left(Z\right)+1\right) \end{array}$$

(A9) $$\begin{array}{cc} \; & =\min\frac{1}{2}\left(2-2x^{T}\mu \left(Z\right)\right) \end{array}$$

(A10) $$\begin{array}{cc} \; & =\min(1-x^{T}\mu \left(Z\right)). \end{array}$$

Noting that the predicted OV semantic embeddings ${\hat{x}}^{*}$ correspond to μ(Z) shows that (A10) is is the cosine distance (31) and thus completes the derivation.
